# Isolated Prostatic Hydatid Cyst: A Case Report

**DOI:** 10.7759/cureus.113938

**Published:** 2026-08-04

**Authors:** Mohammad F Mousa, Ashraf Majali, Bilal A Khawaldeh, Mu'tasem H AlSmadi, Tareq G Alnasser, Ruba A Smadi, Laith A Alshehabat, Mohammed N Al-Ajlouni

**Affiliations:** 1 Urology, Jordanian Royal Medical Services, Amman, JOR; 2 Laboratory Medicine/Microbiology, Jordanian Royal Medical Services, Amman, JOR; 3 General Surgery, Jordanian Royal Medical Services, Amman, JOR

**Keywords:** echinococcus granulosus, hydatid cyst, lower urinary tract obstruction, prostate, transurethral deroofing

## Abstract

Hydatid cystic disease is caused by *Echinococcus granulosus*, a parasite that infects humans and might not spare any organ but the cornea; nevertheless, its occurrence in the pelvis or prostate is extremely rare, with only a few reported cases. We report the case of a 25-year-old gentleman, medically and surgically free, with a negative personal or family history of hydatid cystic disease, who presented to urology clinics with progressive obstructive lower urinary tract symptoms and one episode of urine retention. Pelvic MRI and CT revealed a well-defined cystic lesion in the left prostatic lobe, initially interpreted as a simple cyst. Transurethral deroofing, performed in August 2022 at Prince Hussein Urology Center/Jordanian Royal Medical Services, revealed whitish membranous material suggestive of a hydatid cyst, which was completely evacuated. The postoperative course was uneventful, and there was no other organ involvement upon investigation. Histopathologic study of the cyst showed it to be a hydatid cyst. He was followed up for 24 months with no evidence of local or systemic recurrence and was symptom-free throughout. Isolated prostatic hydatid cysts are extremely uncommon and easily misdiagnosed. In endemic regions, awareness of this entity and careful perioperative assessment are essential for accurate diagnosis. Complete endoscopic evacuation with scolicidal irrigation shows favorable outcomes and can prevent recurrence. In endemic areas, prostatic cystic lesions should raise suspicion for hydatid disease to properly map patients’ management. Moreover, intraoperative suspicion is paramount for definitive diagnosis and successful management.

## Introduction

Cystic echinococcosis is a chronic zoonotic infection caused by the larval stage of *Echinococcus granulosus*, usually transmitted via the feco-oral route from definitive hosts such as dogs and sheep. Liver and lung involvement is seen in 90% of cases, while pelvic or genitourinary involvement is rare, accounting for less than 2-4% of localizations [1]. In genitourinary tract hydatid disease, the kidneys and bladder are most frequently affected, whereas isolated prostatic involvement is very uncommon [2]. As prostatic hydatid cysts have nonspecific clinical and radiologic features, they are usually mistaken for congenital, inflammatory, or neoplastic lesions; hence, prostatic hydatid disease is rarely suspected preoperatively [3]. Most prostatic cysts are diagnosed radiologically via ultrasonography, CT, and MRI; however, the classic radiologic features are often absent in isolated genitourinary disease, rendering imaging modalities nonspecific most of the time [4].

The gold-standard therapy for hydatid cysts, even in rare locations, is surgical with complete evacuation of the cyst while ensuring no spillage to minimize recurrence and anaphylaxis. Such a surgical approach can be minimally invasive and performed endoscopically with favorable outcomes. The transurethral endoscopic approach was first reported with favorable outcomes in 2010 [5]. We present a rare case of an isolated prostatic hydatid cyst in a young male from Amman, Jordan. Our report shows a diagnostic pitfall, emphasizing the importance of pre and intraoperative recognition, and shows the possible efficacy of endoscopic transurethral management in achieving a complete, uneventful recovery from such a disease.

## Case presentation

A 25-year-old man presented to the outpatient urology clinic at the Prince Hussein Urology and Organ Transplantation Center (PHUO), Amman, Jordan, in July 2022, with a three-month history of progressive lower urinary tract obstruction and one episode of acute urinary retention managed by urethral catheterization with a failed trial to void when the urethral catheter was removed as part of our preoperative assessment. Physical examination revealed a firm, non-tender enlargement of the left prostatic lobe, and laboratory results, including complete blood count, renal profile, and urinalysis, were unremarkable.

**Figure 1:**
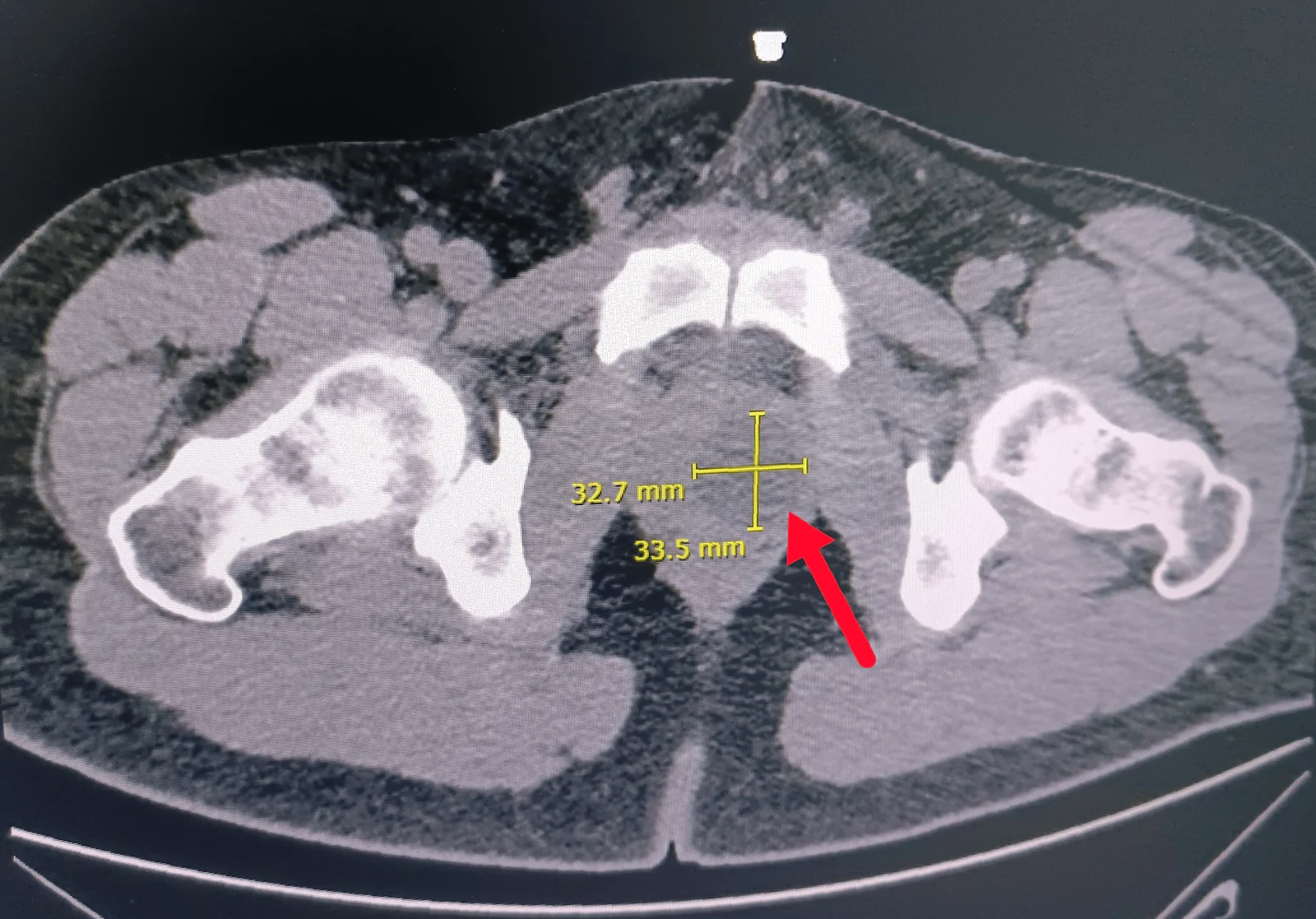
Non-enhanced CT of the pelvis showing the suspicion of a cyst in the left prostatic lobe with its dimensions (red arrow).

Renal and pelvic non-contrast-enhanced CT was performed by the emergency department physicians when the patient presented with acute retention to rule out urethral stones at that time based on their assessment. The CT revealed a thin-walled cyst compressing the bladder without calcifications or solid components, no septations, and no evidence of daughter cysts (Figure 1). In addition, there was no evidence of hepatic cysts.

**Figure 2:**
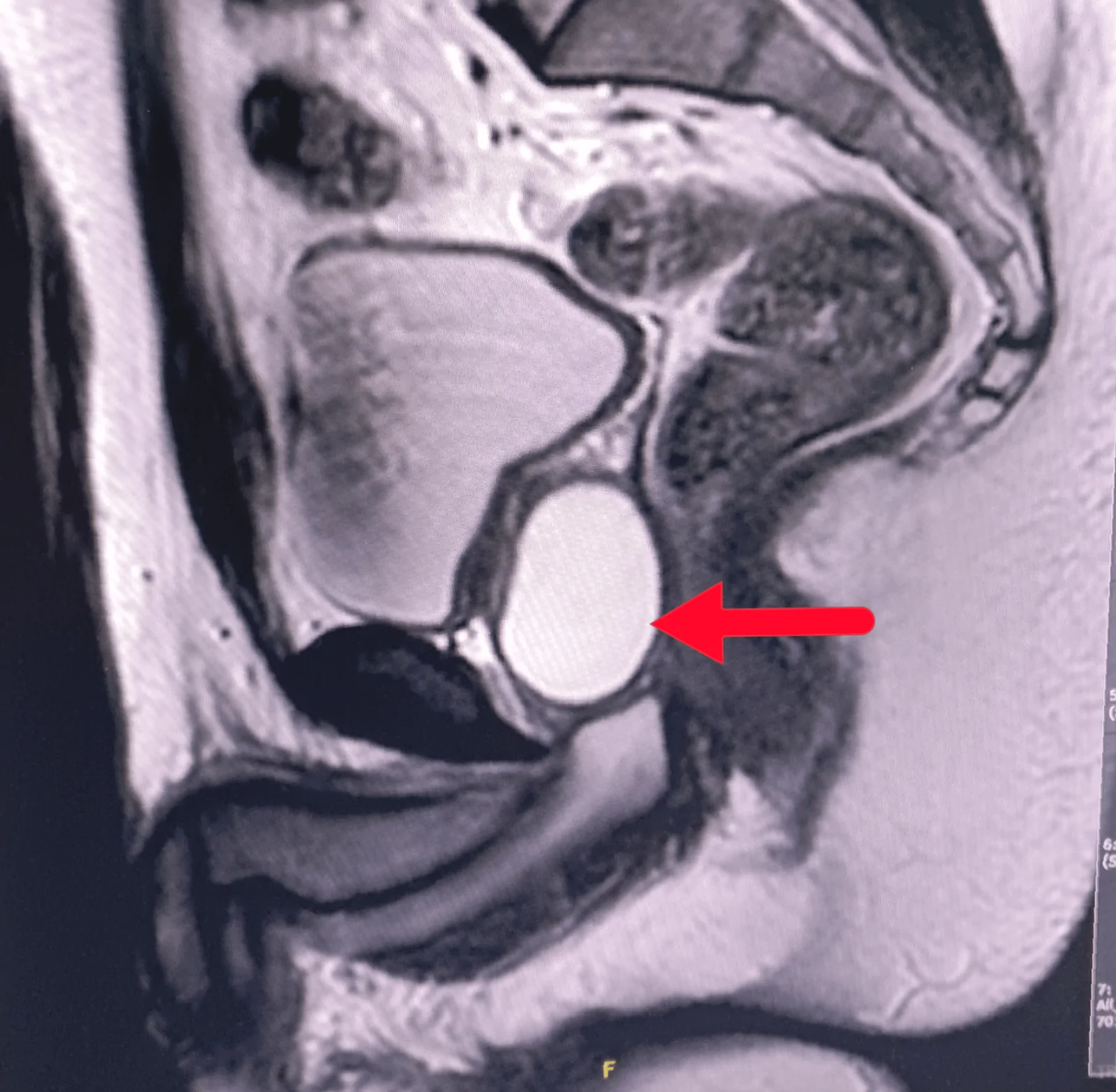
T2 MRI showing a hypeintense left prostatic cyst (red arrow).

Accordingly, pelvic ultrasonography was ordered and showed a well-defined anechoic cystic lesion measuring 32 × 33 mm within the left prostatic lobe. The patient was sent to the Urology clinic with an indwelling urethral catheter. After initial urological assessment, an MRI was ordered and demonstrated a 33 × 46 mm encapsulated cyst with low T1 and high T2 signal intensity, consistent with a benign cystic lesion (Figure 2).

Preoperative seminal fluid analysis was not performed as the patient presented with an indwelling urethral catheter. Preoperative eosinophil count and hydatid serology were also not performed as there was no suspicion of hydatid disease based on the absence of risk factors in the patient’s history. As the radiologic diagnosis was clear for a simple prostatic cyst, our differential diagnosis included Müllerian duct cyst, utricle cyst, and unilateral ejaculatory duct cyst with no suspicion of hydatid cystic disease.

**Figure 3:**
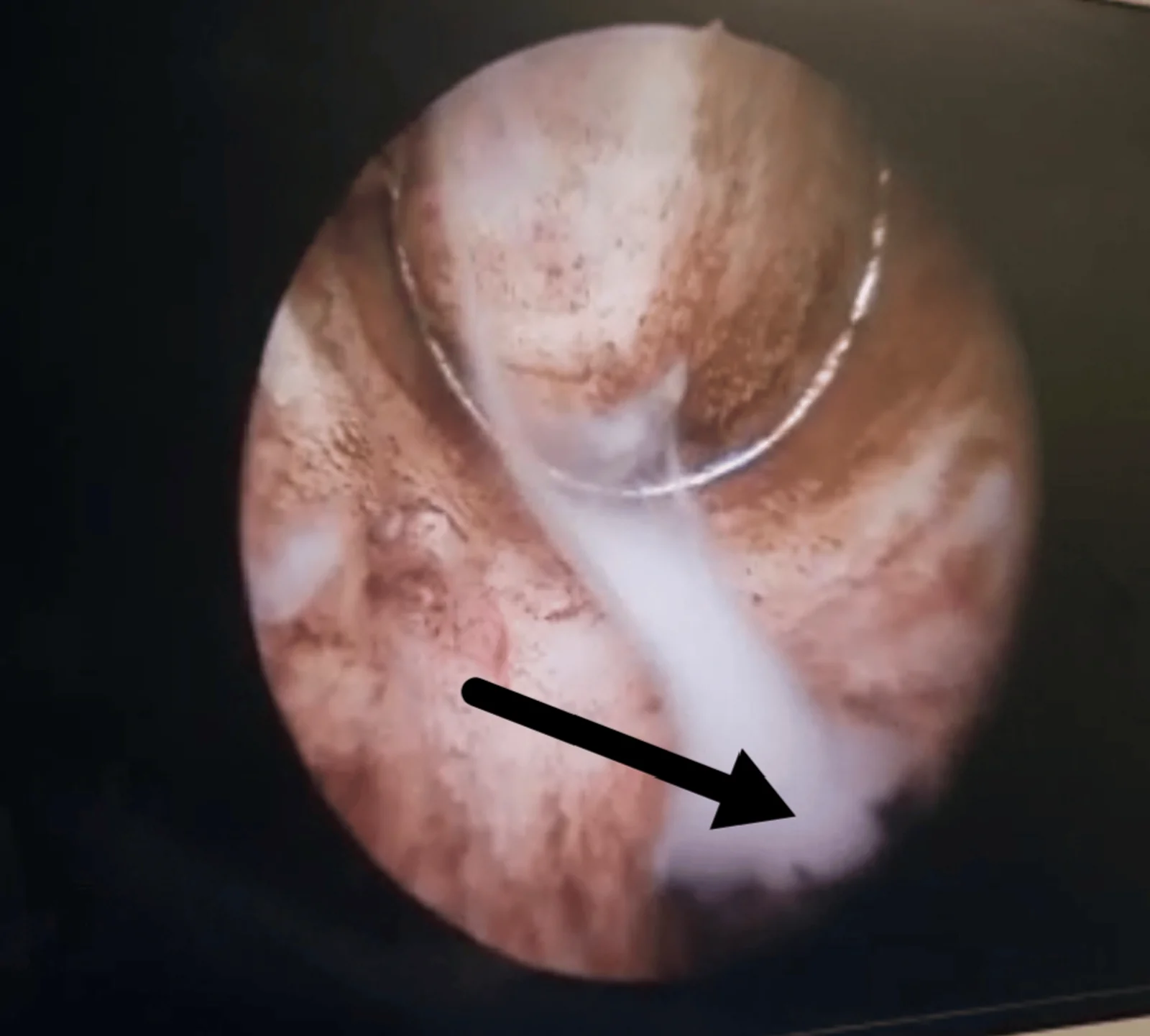
Whitish membranes (germinal layer) (black arrow), appearing upon deroofing of the left lobe prostatic cyst.

In August 2022, under general anesthesia, diagnostic cystoscopy revealed a bulging left prostatic lobe. Transurethral deroofing using a 24-Fr resectoscope exposed whitish membranous material characteristic of a hydatid cyst (Figure 3).

**Figure 4:**
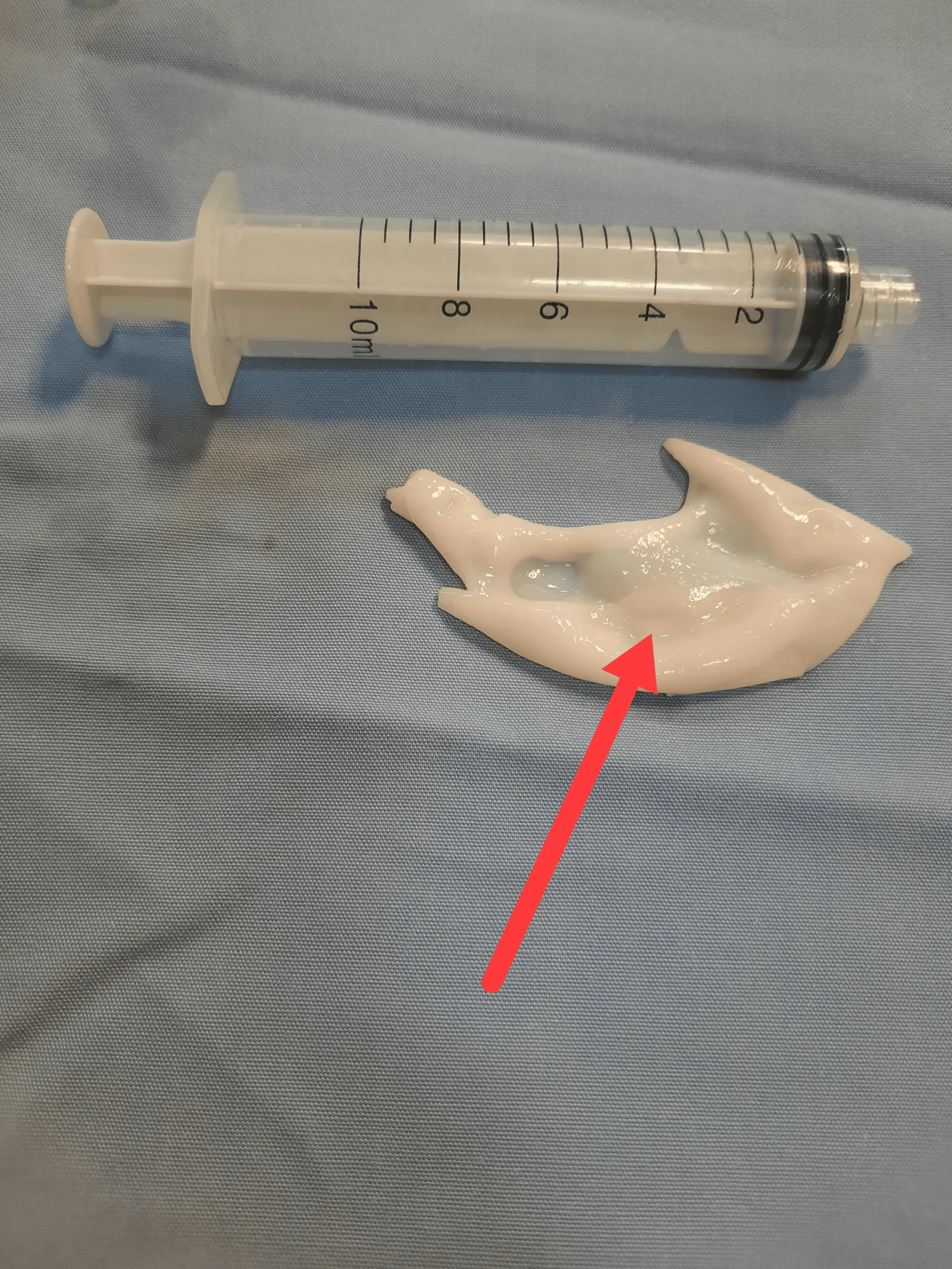
The germinal layer of the hydatid cyst (red arrow) after being evacuated and compared against a 10 mL syringe.

**Figure 5:**
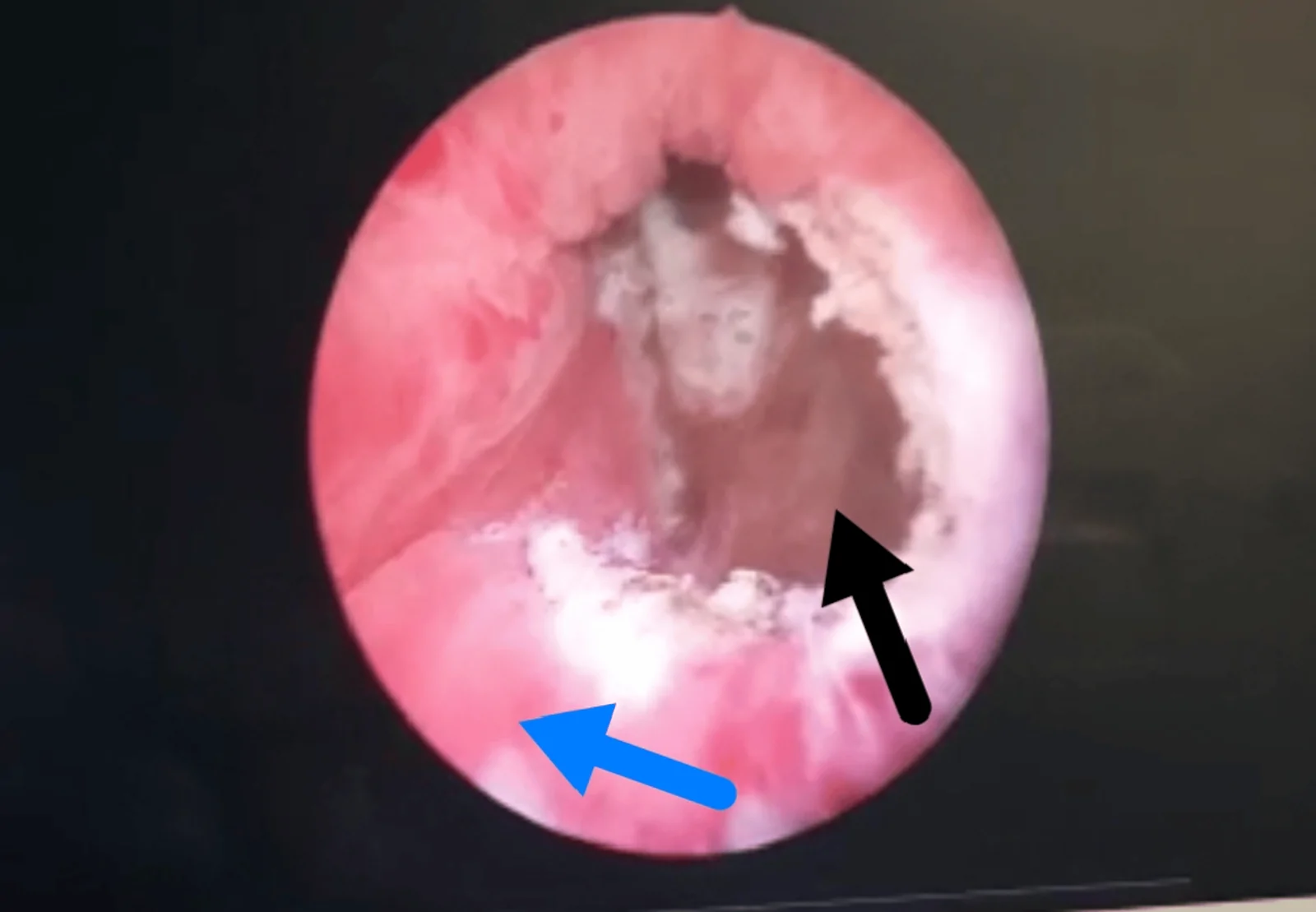
The cavity in the left lobe of prostate (black arrow) with preservation of the verumontanum (blue arrow) after concluding the procedure.

The entire germinal layer was evacuated endoscopically (Figure 4). The cavity was irrigated endoscopically with hydrogen peroxide instilled with the irrigating fluid for five minutes to ensure scolicidal sterilization (Figure 5).

**Figure 6:**
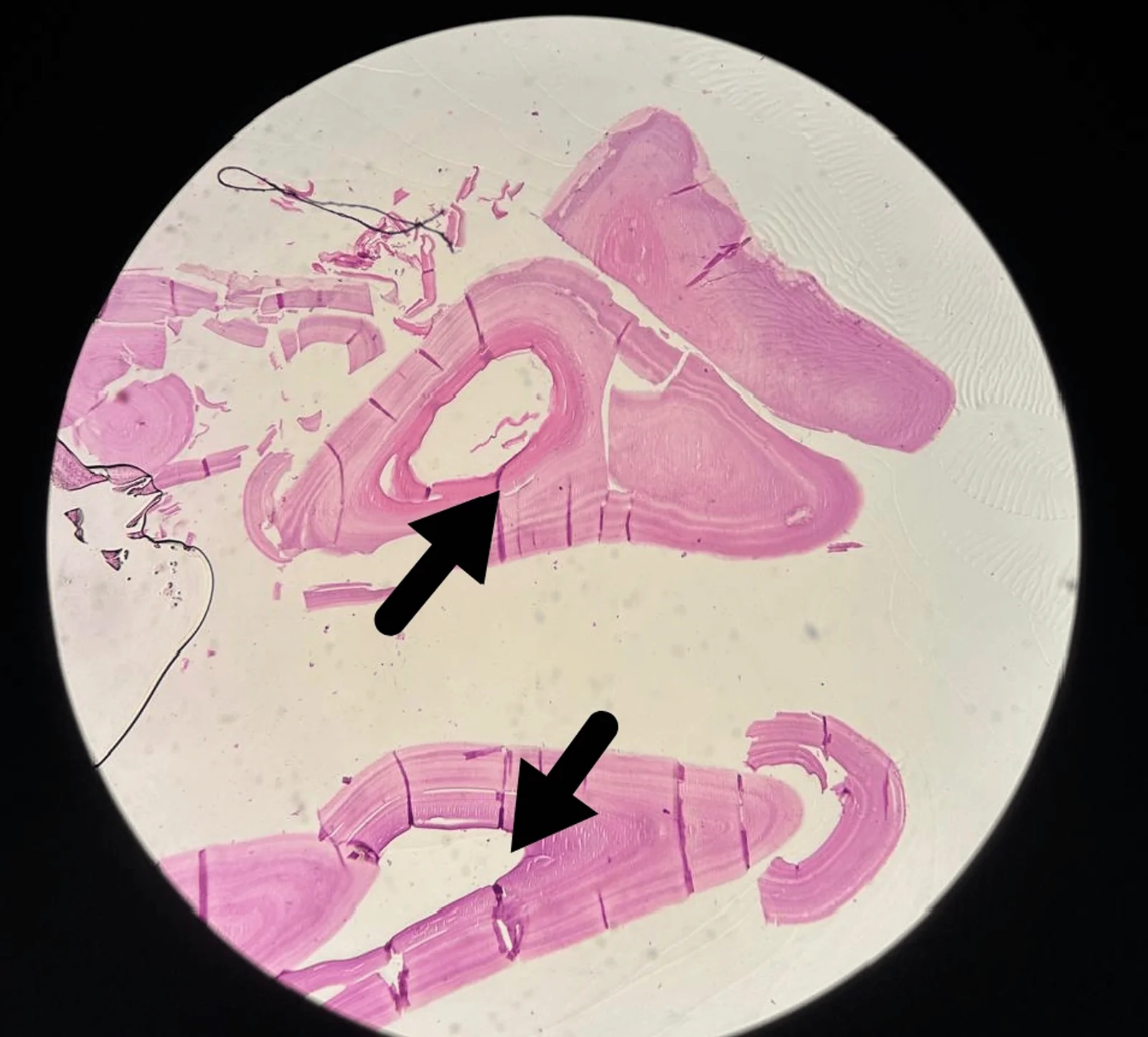
Histopathologic review confirming diagnosis of hydatid cyst (black arrows).

Histopathology confirmed *Echinococcus granulosus* (Figure 6).

The postoperative course was uneventful. The patient urinated freely 48 hours postoperatively, and his urethral catheter was removed; there was no retrograde ejaculation on follow-up. Postoperative evaluation, including eosinophil count, hydatid serology, and chest and abdominal CT scans with contrast, was negative for hydatid and showed no other organ involvement. These investigations were performed 12 hours postoperatively, followed by every six months for 24 months, which were negative throughout. Further, 12 hours postoperatively, the patient was started on albendazole 400 mg oral tablets, twice daily for a total of four cycles (four weeks on treatment and two weeks off). He finished two cycles, and the remaining two cycles were refused by the patient due to poor compliance rather than treatment complications. At 24-month follow-up, the patient remained asymptomatic with no recurrence.

## Discussion

Prostatic hydatid disease is a very rare localization of *Echinococcus granulosus*. Upon literature review, fewer than 20 cases of prostatic hydatid disease have been reported [6]. Prostatic hydatid cysts present with obstructive lower urinary tract symptoms due to urethral and/or bladder neck compression, and such symptoms are also encountered with prostatic congenital cysts or abscesses [7]. Prostatic hydatid cysts mostly lack the pathognomonic radiologic features of hydatid disease seen in hepatopulmonary sites, mainly due to absence of daughter cysts and calcifications, leading to interpretation of such prostatic cysts as congenital [8]. Serological tests have variable sensitivity based on disease burden and are mostly negative in isolated pelvic disease [9].

Open or minimally invasive surgical evacuation is the mainstay of treatment. The goal is to completely evacuate the cyst contents while avoiding rupture and spillage, which may cause recurrence or anaphylaxis [10]. Transurethral deroofing offers a minimally invasive approach for intraprostatic cysts with preservation of urinary and sexual function; however, long-term follow-up is warranted as recurrence can occur years following surgical treatment [11]. Several scolicidal agents are used to irrigate around the cysts during operative deroofing, including hypertonic saline, povidone-iodine, hydrogen peroxide, ethanol, and silver nitrate. These agents vary in safety for use and potential local or systemic toxicity, warranting the choice of agent to be based on experience with their routine use intraoperatively [12]. Although rare, hydatid disease of the prostate is being increasingly reported in endemic areas, necessitating a level of suspicion of hydatid disease in patients presenting with isolated prostatic cysts to ensure appropriate therapy for such patients [13].

The first case of isolated prostatic hydatid was reported in 1975 by Houston [14]. Similar to our case, the patient was a young 23-year-old gentleman presenting with acute urinary retention and failure of urethral catheterization. Cystoscopy was performed, revealing a large prostatic urethral cyst which was deroofed; obstruction was relieved, but they encountered a more significant pitfall, as the diagnosis of hydatid was not fulfilled intraoperatively and the patient urinated the germinal layers postoperatively.

Additionally, Sallami et al. (2010) reported an isolated prostatic cyst causing progressive obstructive lower urinary tract symptoms rather than acute obstruction in a 46-year-old gentleman [5]. Unlike our case, the diagnosis was made preoperatively. Cystoscopic deroofing of the cyst with extraction of germinal layers was performed, similar to our technique, with use of the same scolicidal irrigation agent. There were no complications; however, postoperatively, the patient did not undergo albendazole therapy. Nonetheless, he remained free of recurrence during the 16-month follow-up period.

Furthermore, a case of prostatic hydatid cyst with spontaneous rupture and subsequent development of stones within the hydatid cyst cavity was reported by Nouira et al. (2006) [15]. The patient was a 52-year-old gentleman presenting with irritative and obstructive lower urinary tract symptoms and multiple opaque stones within the prostatic urethra. Cystoscopy found an old cystic cavity with stones in the prostate. The stones were disintegrated, and transurethral resection of the prostate along with the cavity was performed, which was later shown to contain hydatid disease within the resected cyst cavity on histopathology. Unlike our case, this case shows that the cyst may have ruptured before the cystoscopic intervention. Intraoperative diagnosis was not clear with no use of scolicidal irrigation. Despite this, the patient had no complications and suffered no recurrence.

## Conclusions

Isolated prostatic hydatid cyst is very rare and thus easily mistaken for benign or congenital prostatic cysts, especially when the history lacks risk factors and the radiologic findings are simple and non-suggestive, as in our case. Definitive preoperative diagnosis can be not straightforward due to the absence of specific radiologic or serologic findings. Intraoperative identification of hydatid membranes remains the most reliable diagnostic clue. Complete endoscopic evacuation followed by scolicidal irrigation can be a safe, minimally invasive, and possibly curative approach, yet further experience needs to be reported with such prostatic hydatid cysts to supplement our findings; however, unfortunately, the disease entity is very rare. In endemic regions, maintaining a high index of suspicion for hydatid disease is essential to avoid a diagnostic pitfall, such as in our case, allowing to properly counsel and prepare patients both pre and intraoperatively.
